# Social Media Use and Mental Health in Young Adults of Greece: A Cross-Sectional Study

**DOI:** 10.32872/cpe.4621

**Published:** 2022-06-30

**Authors:** Epameinondas Leimonis, Katerina Koutra

**Affiliations:** 1 Department of Psychology, School of Social Sciences, University of Crete, Rethymno, Greece; Philipps-University of Marburg, Marburg, Germany

**Keywords:** social media, mental health, self-esteem, depressive symptoms, young adults

## Abstract

**Background:**

Social media use has vastly increased during the past few years, especially among young adults. Studies examining the relationship of social media use with mental health have yielded mixed findings. Additionally, such studies are extremely limited in Greece. The present study aimed to investigate the association between social media use, depressive symptoms and self-esteem among Greek young adults.

**Method:**

A total of 654 individuals (50.5% male) aged 18-30 years (Μ *=* 23.62, SD *=* 2.71) completed self-reported questionnaires regarding social media use, depressive symptoms and self-esteem.

**Results:**

Increased daily use of YouTube (more than five hours) showed a significant association with higher depressive symptomatology, b = 2.99, 95% CI [.78, 5.20], p = .008, while daily use of Facebook between two and five hours was related to significantly higher self-esteem, b = 1.61, 95% CI [.78, 2.44], p < .001, after adjusting for participants’ gender, age, educational level and employment status. The association of increased daily use of YouTube with depressive symptoms was more pronounced in males than in females. Moreover, self-reported active use of Facebook and Instagram were linked with significantly lower depressive symptoms and higher self-esteem compared to passive involvement.

**Conclusion:**

The results suggest that social media use is closely related to self-esteem and depressive symptomatology in young adults. These findings may contribute to a deeper clinical understanding of the association between electronic social networking and mental health.

## Highlights

- Increased daily time spent in YouTube (more than five hours) was significantly associated with higher depressive symptomatology.
- YouTube daily use of more than five hours showed a stronger association with depressive symptoms for males than for females.
- Daily use of Facebook between two and five hours was related to significantly higher self-esteem.
- Self-reported active use of Facebook and Instagram were linked with significantly lower depressive symptoms and higher self-esteem compared to passive involvement.

Electronic social networking is undoubtedly a worldwide technological phenomenon with various extensions in modern human life. Through social media people are able to communicate and interact with each other, while they also have the opportunity to develop and share electronic data (Ellison et al., 2007; Kaplan & Haenlein, 2010). During the past few years, social media have become extremely popular, especially among young adults (Pew Research Center, 2015). Specifically, 90% of U.S. adults aged 18-29 are frequent users of at least one online social network, while YouTube and Facebook are the most popular platforms (Pew Research Center, 2019). In Greece, almost 60% of total population are involved in social media use (Hootsuite & We Are Social, 2020), while young adults constitute the vast majority of users (Belenioti, 2015). Similarly to other countries, Facebook and YouTube are the two most widely used social networks in Greece (Drosos et al., 2015).

Due to the fast and constantly increasing penetration of social media in everyday life, their association with mental health has gained considerable attention within the scientific community. Recent studies have provided mixed results, either indicating harmful effects of social networks on users’ psychological well-being (e.g. Rasmussen et al., 2020; Sujarwoto et al., 2019) or suggesting non-significant associations (e.g. Coyne et al., 2020). Social media users facing mental health difficulties appear to experience both benefits, such as easy social interaction, access to peer support, increased involvement in various services, and negative consequences, including increased symptoms and exposure to aggressive online behaviour (Naslund et al., 2020). Overall, international research has showed that social networking can affect mental health both positively and negatively (Sharma et al., 2020).

Depression is a mood disorder considered as the primary cause of disability and one of the most common causes of death between the ages of 15 and 29 years, since it is responsible for more than 800,000 annual suicides (World Health Organization, 2020). The relationship between social media use and depression has been in the spotlight of research for about a decade, with many studies suggesting that increased involvement in online social networking is associated with higher levels of depressive symptomatology (Ivie et al., 2020; McDougall et al., 2016; Pantic et al., 2012; Woods & Scott, 2016). Elongated daily time spent in social media has been linked to higher odds of depression in young adults (Lin et al., 2016), while the use of multiple social networking platforms has been related to increased depressive symptoms (Primack et al., 2017). In a recent study, the reduction of time spent in social networks was linked to significantly less negative mental health outcomes, including less depressive symptoms, in young adults (Hunt et al., 2018). Social comparison has often mediated the aforementioned associations (e.g. Brown & Tiggemann, 2016; Lup et al., 2015), while envy has displayed a mediating effect on the relationship between social comparison and adult users’ depressive symptoms (Wang et al., 2020). Finally, addiction to social media has been significantly associated with depression (Donnelly & Kuss, 2016).

Despite what appears to be evidence for a negative association between social media and depressive symptomatology, other studies have showed that social media use can have positive effects on individuals’ well-being. Communication and interaction through these networks have been found to contribute to an increase in social capital and, thus, a reduction in depressive symptoms (Bessière et al., 2010; de la Peña & Quintanilla, 2015; Ellison et al., 2007). Platforms such as Snapchat, Twitter, Instagram and Facebook provide opportunities for participation in positive social interactions among various sources of social support, which can alleviate depressive symptoms (Bessière et al., 2010). Moreover, these platforms may help people form connections with other individuals suffering from stigmatised health conditions such as depression (Merolli et al., 2014). In a similar vein, active use of social networks (e.g. sharing content and communicating with other users) has been linked with decreased depressive symptoms and pertinent outcomes compared to passive use (e.g. avoiding posting new content, visiting other users’ profiles and following their posts) (Escobar-Viera et al., 2018; Verduyn et al., 2015). In general, the relationship between social media use and depressive symptoms appears to be complicated and influenced by various individual and psychosocial factors (Baker & Algorta, 2016).

Previous studies have suggested a negative association between depressive symptoms and self-esteem (Conti et al., 2014; Franck et al., 2007). Self-esteem is defined as the subjective way in which individuals perceive their personal value (MacDonald & Leary, 2012). With respect to the association between social media use and self-esteem, findings appear to be mixed. Specifically, recent research indicates that increased involvement in social networks is linked to lower self-esteem in adolescents and young adults (e.g. Bergagna & Tartaglia, 2018; Vogel et al., 2014; Woods & Scott, 2016). On the contrary, some researchers have found that social media use is related to higher self-esteem (e.g. Gonzales & Hancock, 2011; Wilcox & Stephen, 2013). Two mechanisms that seem to explain or mediate such relationships include the kind of feedback that users receive from online social networks (Valkenburg et al., 2017; Valkenburg et al., 2006) as well as social comparison (Bergagna & Tartaglia, 2018; Vogel et al., 2014). Moreover, cyberbullying through social media has been related to lower self-esteem levels (Palermiti et al., 2017).

Little research has been conducted so far regarding electronic social networking and aspects of mental health in the Greek population. Recent findings suggest that almost 34% of Greek adolescent users report intense activity in social networks, while approximately 10% display problematic use, which refers to addictive behaviour (Boer et al., 2020). With regard to Greek young adults, excessive social media use has been linked with higher levels of loneliness and decreased life satisfaction (Vasilikou, 2016). In addition, excessive use of social networking sites has been significantly associated with personality factors, such as neuroticism, along with increased depressive symptomatology in young Greeks (Giota & Kleftaras, 2013). Although little research has been conducted, recent findings have suggested that the frequency of social media use, such as Facebook, is not associated with self-esteem in adolescents (Botou & Marsellos, 2018). However, cyberbullying has been related to low self-esteem in university students of Greece (Giovazolias & Malikiosi-Loizos, 2016).

There is considerable evidence that social media use is linked with mental health, including depressive symptomatology and self-esteem, positively or negatively (e.g. Bessière et al., 2010; Lin et al., 2016). However, approximately half of previous studies have examined social media use in general (Schønning et al., 2020), and many of them as a single variable, without providing results regarding the use of different platforms (e.g. Escobar-Viera et al., 2018; Lin et al., 2016; Woods & Scott, 2016). Studies assessing the use of specific social networks in relation to mental health outcomes have focused mainly on Facebook (e.g. Bergagna & Tartaglia, 2018; Wilcox & Stephen, 2013). Furthermore, the amount of Greek data concerning the relationship between social networks and human behaviour is extremely limited. Taking into consideration the above-mentioned gap in the literature, the aim of the present study was to investigate the association of different popular social media with self-esteem and depressive symptoms in a large Greek sample of emerging adults. We hypothesised that increased time of social media use was significantly associated with higher depressive symptoms and lower self-esteem in young adults. We, also, hypothesised that active social media users would have lower symptoms of depression and higher self-esteem compared to passive social media users.

## Method

### Participants

To be eligible for inclusion in the study, participants had to meet the following criteria: (i) to be between 18 and 30 years old, (ii) to use at least one electronic social network, and (iii) to have a good understanding of the Greek language. The sample included 654 young adults (50.5% male and 49.5% female) aged 18-30 years (*Μ* = 23.62, *SD* = 2.71). The vast majority of them were Greek (98.9%) and residents of urban areas (94.3%). The sample composition of participants’ highest level of education completed was: 58.3% high school/vocational education and training, 33.9% university/college degree, and 7.8% postgraduate studies. Furthermore, 98.6% of the participants were unmarried and 59.9% reported a low monthly income (up to 500€). Regarding employment status, 59.5% of participants were not working. The sociodemographic characteristics of the participants are presented in Table 1.

**Table 1:** Sociodemographic Characteristics of Participants and Associations With Depressive Symptoms and Self-Esteem (N = 654)

Sociodemographic variables			Depressive symptoms			Self-esteem		
	*N*	%	*M*	*SD*	*p*	*M*	*SD*	*p*
Gender					.330			.140
Male	330	50.5	9.24	7.98		30.86	4.59	
Female	324	49.5	9.85	8.18		30.31	4.97	
Nationality					.514			.079
Greek	647	98.9	9.50	7.99		30.62	4.79	
Other	7	1.1	13.29	14.44		27.43	3.74	
Place of origin					.086			.496
Urban	527	80.6	9.28	8.09		30.65	4.78	
Rural	127	19.4	10.65	7.96		30.33	4.81	
Place of residence					.167			.483
Urban	617	94.3	9.43	8.11		30.62	4.81	
Rural	37	5.7	11.32	7.27		30.05	4.43	
Educational level					.396			.610
High school/V.E.T.	381	58.3	9.85	8.25		30.67	4.61	
University/College	222	33.9	8.94	7.49		30.36	5.01	
Postgraduate studies	51	7.8	9.88	9.19		30.98	5.09	
Employment status					.191			.448
Working	265	40.5	10.04	8.23		30.76	5.03	
Non-working	389	59.5	9.20	7.96		30.47	4.62	
Net monthly income					.396			.051
0 € - 500 €	392	59.9	9.70	8.35		30.29	4.73	
> 500 €	249	38.1	9.15	7.49		31.05	4.86	
Marital status					.097			.814
Unmarried	645	98.6	9.60	8.10		30.59	4.81	
Married	9	1.4	5.11	5.09		30.78	2.28	
	*M*	*SD*	*Min-max*	*r*	*p*		*r*	*p*
**Participants' age**	23.62	2.71	18–30	-.043	.273		.058	.139

*Note*. *t*-test and ANOVA were used for differences between continuous variables; Pearson’s *r* was used for correlation between continuous variables.

### Measures

#### Sociodemographic Characteristics

Sociodemographic variables included participants’ gender, age, nationality (Greek or other), place of origin and residence (urban vs. rural), educational level that each participant had completed (high school/vocational education and training, university/college degree, postgraduate studies), employment status (working vs. non-working), marital status (unmarried vs. married), and net monthly income (0-500€ vs. 501€ and above).

#### Social Media Use

We assessed participants’ social media involvement based on daily time use and type of user (active/passive user), influenced by recent studies (Escobar-Viera et al., 2018; Lin et al., 2016). Due to the lack of Greek standardised psychometric tools concerning the above-mentioned variables, we designed a brief self-reported questionnaire consisting of three items based on a previous study about internet and social media use in relation to consumer behaviour (Koutsogiannopoulou, 2013). First, participants were asked whether they had been using social media (“Do you use social media?”), responding to an alternative form question (“yes” or “no”), and provided estimates about their daily use of specific popular platforms, including Facebook, Twitter, Instagram, YouTube, Tumblr, LinkedIn, Skype, and blogs. Four response choices were offered in a Likert-type scale (“not at all”, “less than two hours”, “two to five hours”, “more than five hours”). Moreover, individuals were asked to characterise their involvement on each one of these networks as “passive” or “active” after explanation of these two terms was offered. Specifically, passive users were considered those who maintained activities such as limited communication and sharing of electronic content, along with passive following of other users’ posts. Conversely, individuals who engaged more in interaction with others and sharing of various types of content were considered as active users. To ensure participants’ best comprehension of these patterns of activity, we used definitions and examples based on previous studies (e.g. Escobar-Viera et al., 2018).

#### Self-Esteem

Self-esteem was measured by means of the Rosenberg Self-Esteem Scale (RSES; Rosenberg, 1965). The RSES consists of 10 items in form of statements which are related to self-esteem (e.g. “I feel I do not have much to be proud of”). Of these statements, five are positively graded (1, 2, 4, 6, 7) and five are negatively graded (3, 5, 8, 9, 10). Each individual is asked to respond to a four-point Likert scale, ranging from 1 (“strongly disagree”) to 4 (“strongly agree”). Total score ranges from 10 to 40 with higher scores indicating higher levels of self-esteem. High self-esteem scores suggest that individuals have self-respect and consider him or herself worthy. Low self-esteem scores suggest an unfavorable opinion of oneself and self-dissatisfaction. The scale has been translated and validated for the Greek population by Tsagarakis et al. (2007). In the present study, the scale demonstrated satisfactory internal consistency (α = .84).

#### Depressive Symptoms

Depressive symptomatology was measured using the Beck Depression Inventory-II (BDI-II; Beck et al., 1996). The BDI-II is a 21-item, self-report rating inventory that measures characteristic attitudes and symptoms of depression (Beck et al., 1996), while it taps major depression symptoms according to diagnostic criteria listed in the Diagnostic and Statistical Manual for Mental Disorders (American Psychiatric Association, 2000). Each item is assessed on a four-point scale (0–3). The total score indicates whether the individual presents a mild, moderate or major depression (possible range 0-63). The BDI-II has been translated and validated in Greek by Giannakou et al. (2013). In the present study, the scale showed satisfactory internal consistency (α = .86).

### Procedure

Participants were recruited through the research team contacting different academic departments, and disseminating a web link to each student which provided details of the study. Moreover, non-university student participants were recruited via online posts at social media groups. Information about anonymous and voluntary participation was provided to participants prior to data collection. Confidentiality was assured and informed consent was obtained from the participants. Finally, participants were given written instructions for filling out the questionnaires and were informed about the estimated time needed for completing the measures (approximately 15 minutes). The study was conducted in accordance with the ethical standards delineated in the 1964 Declaration of Helsinki and its later amendments or comparable ethical standards. Ethical approval was granted by the Psychology Department’s Research Ethics Committee. Informed consent was obtained from all individual participants included in the study.

### Data Analysis

With regard to descriptive data, we computed percentages of sociodemographic variables, daily time use of social networks and type of social media use (active and passive use). In terms of descriptive indices, we calculated means (*M*) and standard deviations (*SD*) in order to better frame our results. Social media use was not assessed as a single numeric variable, since we focused our analyses on daily time of use (less than two hours, two to five hours, more than five hours) and self-reported type of use (passive vs. active). Considering that YouTube, Facebook and Instagram showed by far the highest percentages of daily users, our analyses were focused on the specific platforms.

*t*-test and ANOVA were used for the comparison of independent groups. Specifically, we employed one-way ANOVAs, including post-hoc comparisons using Tukey, to separately investigate differences in depressive symptomatology and self-esteem between categories regarding daily use of YouTube, Facebook and Instagram (less than two hours, two to five hours, more than five hours). Moreover, we used independent samples *t*-tests to assess differences in depressive symptoms and self-esteem between categories concerning self-reported type of YouTube, Facebook and Instagram use (active and passive). Pearson’s *r* correlation coefficient was used to estimate the strength of the association between self-esteem and depressive symptoms. Multiple linear regression models were also implemented to further and separately examine the associations of YouTube, Facebook and Instagram use (daily time and type of use) with depressive symptoms and self-esteem, after adjusting for confounding variables. Potential confounders related with both the outcome and/or the independent variables in group comparisons with a *p*-value < .2 were included in the models. Therefore, each model was adjusted for participants’ gender, age, educational level and employment status, while estimated associations were described in terms of *b*-coefficients (beta). We were also able to examine effect modification stratifying by gender. For interaction terms, we considered *p*-value < .05 as nominally significant. All other hypotheses testing was conducted assuming a .05 significance level and a two-sided alternative hypothesis. All analyses were conducted by means of the IBM SPSS Statistics 26 software.

## Results

### Prevalence of Social Media Use

In terms of daily time of engagement in social media, YouTube was the most popular platform with respect to users (97.6%), followed by Facebook (93.3%), Instagram (81.8%), blogs (15.9%), Skype (12.1%), LinkedIn (10.9%), Twitter (4.3%) and Tumblr (3.1%). Only 17.7% of the participants reported using some other platform except for the specific ones. Overall, 99.4% of the participants were found to use more than one platform daily. Concerning the self-reported type of social media use, 65.8% of daily YouTube users reported being passive, while 51.3% of daily Facebook users mentioned being active. Instagram demonstrated the highest self-reported active online engagement (75%). Additionally, more than 50% of daily users of blogs, Skype, LinkedIn and Twitter stated passive involvement in these platforms, whereas half of Tumblr everyday users reported being active.

### Associations Between Sociodemographic Characteristics and Study Variables

Tables 1 and 2 show associations between participants’ sociodemographic characteristics and the outcome variables of our study. Specifically, non-significant relationships were found between sociodemographic variables, depressive symptomatology and self-esteem at a level of *p*-value < .05 (Table 1). According to Table 2, significant differences between men and women were found in terms daily use of YouTube, χ^2^(2, *N* = 638) = 10.11, *p* = .006, and Instagram, χ^2^(2, *N* = 535) = 11.74, *p* = .003, as well as self-reported type of Instagram use, χ^2^(1, *N* = 535) = 9.20, *p* = .002. In addition, participants’ age was significantly related to all social media use variables, while educational level showed significant associations with self-reported type of YouTube, Facebook and Instagram use. Employment status was linked with daily use of YouTube, χ^2^(2, *N* = 638) = 11.17, *p* = .004, along with self-reported type of YouTube, Facebook and Instagram use. Individuals’ net monthly income was significantly associated with daily use of YouTube, χ^2^(2, *N* = 625) = 6.59, *p* = .037.

**Table 2:** Participants’ Sociodemographic Characteristics and Social Media Use (N = 654)

Sociodemographic variables	Social media daily use						Social media user type					
	YouTube^a^		Facebook^a^		Instagram^a^		YouTube^b^		Facebook^b^		Instagram^b^	
	**χ^2^**	***p* ^c^**	**χ^2^**	***p* ^c^**	**χ^2^**	***p* ^c^**	**χ^2^**	***p* ^c^**	**χ^2^**	***p* ^c^**	**χ^2^**	***p* ^c^**
Gender	10.11	.006**	4.50	.105	11.74	.003**	3.79	.053	1.51	.219	9.20	.002**
Nationality	1.58	.455	1.02	.602	1.05	.592	.00	.965	1.99	.160	.00	1.000
Place of origin	2.48	.289	.01	.995	1.38	.501	.04	.837	.69	.406	1.36	.244
Place of residence	2.07	.356	.16	.923	1.20	.548	.22	.638	.47	.494	.70	.404
Educational level	5.41	.247	6.18	.186	5.09	.278	40.78	< .001***	18.02	< .001***	16.91	< .001***
Employment status	11.17	.004**	1.26	.532	1.55	.461	9.54	.002**	4.78	.029*	4.22	.040*
Net monthly income	6.59	.037*	1.72	.422	1.81	.405	1.04	.307	2.40	.121	3.11	.078
Marital status	2.19	.700	1.00	.911	6.14	.189	.62	.733	1.68	.431	5.72	.057
	*F*	*p* ^d^	*F*	*p* ^d^	*F*	*p* ^d^	*t*	*p* ^e^	*t*	*p* ^e^	*t*	*p* ^e^
Participants' age	3.78	.023*	5.33	.005**	4.78	.009**	4.77	< .001***	3.93	< .001***	4.94	< .001***

^a^Includes daily use of less than 2 hours, 2-5 hours, and more than 5 hours. ^b^Includes self-reported active user and passive user. ^c^*p* value derived using chi-square analysis. ^d^*p*-value derived using one-way ANOVA. ^e^*p*-value derived using independent samples *t*-test.

*p < .05. **p < .01. ***p < .001.

### Differences in Depressive Symptomatology and Self-Esteem by Social Media Groups

Table 3 shows differences in depressive symptomatology and self-esteem by social media groups. One-way ANOVA results indicated a statistically significant difference in the mean score of self-esteem between the different categories of Facebook daily use, *F*(2, 607) = 6.88, *p* = .001, η^2^ = .02. Particularly, a post-hoc Tukey test showed that individuals who reported two to five hours of Facebook everyday use had significantly higher self-esteem (*N* = 185, *M* = 31.62, *SD* = 4.87) compared to those who had been using Facebook for less than two hours daily (*N* = 365, *M* = 30.04, *SD* = 4.51, *p* = .001).

**Table 3:** Group Differences in Depressive Symptomatology and Self-Esteem by Social Media Use (N = 654)

Social media use		Depressive symptomatology^a^					Self-esteem^a^				
	*N*	*M*	*SD*	*F*	*p* ^b^	η^2^	*M*	*SD*	*F*	*p* ^b^	η^2^
YouTube daily use				2.94	.053	.01			.35	.703	.00
Less than 2 hours	316	9.23	7.59				30.63	4.69			
2-5 hours	262	9.33	7.87				30.73	4.68			
More than 5 hours	60	11.90	10.31				30.15	5.67			
Facebook daily use				.94	.391	.00			6.88	.001**	.02
Less than 2 hours	365	9.59	7.98				30.04	4.51			
2-5 hours	185	9.03	7.60				31.62	4.87			
More than 5 hours	60	10.62	8.56				30.72	5.33			
Instagram daily use				.74	.477	.00			1.00	.370	.00
Less than 2 hours	215	9.37	8.07				30.33	4.59			
2-5 hours	254	9.69	7.91				30.95	4.91			
More than 5 hours	66	10.74	8.35				30.86	5.30			
	*N*	*M*	*SD*	*t*	*p* ^c^	*d*	*M*	*SD*	*t*	*p* ^c^	*d*
YouTube user type				.47	.641	.04			-1.56	.119	.13
Active users	218	9.32	7.97				31.03	4.96			
Passive users	420	9.63	8.05				30.41	4.68			
Facebook user type				2.55	.011*	.21			-4.30	< .001***	.35
Active users	313	8.73	7.27				31.18	4.75			
Passive users	297	10.36	8.51				29.75	4.61			
Instagram user type				3.33	.001**	.32			-2.32	.021*	.23
Active users	401	9.03	7.61				30.97	4.81			
Passive users	134	11.67	8.91				29.86	4.84			

^a^Depressive symptomatology and self-esteem were treated as continuous numeric variables. ^b^*p*-value derived using one-way ANOVA. ^c^*p*-value derived using independent samples *t*-test.

*p < .05. **p < .01. ***p < .001.

According to *t*-test results, a significant mean difference was found between self-reported active Facebook users (*N* = 313, *Μ* = 8.73, *SD* = 7.27) and passive Facebook users (*N* = 297, *Μ* = 10.36, *SD* = 8.51), *t*(583) = 2.55, *p* = .011, *d* = .21. There was also a significant difference in depressive symptoms between self-reported active Instagram users (*N* = 401, *Μ* = 9.03, *SD* = 7.61) and passive Instagram users (*Ν* = 134, *Μ* = 11.67, *SD* = 8.91), *t*(533) = 3.33, *p* = .001, *d* = .32. A significant difference was observed in self-esteem between self-reported active Facebook users (*Ν =* 313, *Μ =* 31.38, *SD* = 4.75) and passive Facebook users (*Ν =* 297, *Μ =* 29.75, *SD* = 4.61), *t*(608) = -4.30, *p* < .001, *d* = .35. Likewise, there was a significant difference in self-esteem between active Instagram users (*Ν =* 401, *Μ =* 30.97, *SD* = 4.81) and passive Instagram users (*Ν =* 134, *Μ =* 29.86, *SD* = 4.84), *t*(533) = -2.32, *p* = .021, *d* = .23. Finally, self-esteem was significantly and negatively correlated with depressive symptomatology, *r*(652) = -.55, *p* < .001.

### Multivariable Associations of Social Media Daily Use and User Type With Depressive Symptomatology and Self-Esteem

According to multiple linear regression results (Table 4), daily YouTube use of more than five hours was associated with significantly higher BDI-II scores, after controlling for gender, age, educational level and employment status, *b* = 2.99, 95% CI [.78, 5.20], *p* = .008. The model explained 2% (adjusted 1.1%) of the variance in BDI-II scores (*R*^2^ = .02). Daily use of Facebook and Instagram showed non-significant associations with depressive symptoms.

**Table 4:** Adjusted Associations of Social Media Daily Use and User Type With Depressive Symptomatology and Self-Esteem (N = 654)

Models^a^	Depressive symptomatology^b^				Self-esteem^b^			
	*B^c^*	*SE B*	95% CI^c^	*p*	*B^c^*	*SE B*	95% CI^c^	*p*
YouTube daily use								
2-5 hours vs. < 2 hours^d^	.30	.67	[-1.01, 1.62]	.652	.09	.40	[-.70, .88]	.819
> 5 hours vs. < 2 hours^d^	2.99	1.13	[.78, 5.20]	.008**	-.45	.68	[-1.78, .89]	.512
Facebook daily use								
2-5 hours vs. < 2 hours^d^	-.60	.71	[-1.99, .80]	.402	1.61	.42	[.78, 2.44]	< .001***
> 5 hours vs. < 2 hours^d^	.70	1.11	[-1.47, 2.87]	.525	.83	.66	[-.46, 2.12]	.205
Instagram daily use								
2-5 hours vs. < 2 hours^d^	.01	.75	[-1.46, 1.47]	.995	.82	.45	[-.06, 1.70]	.068
> 5 hours vs. < 2 hours^d^	1.04	1.13	[-1.17, 3.26]	.356	.81	.68	[-.53, 2.14]	.236
YouTube user type								
Active vs. passive^d^	-.60	.69	[-1.96, .75]	.383	.64	.41	[-.17, 1.45]	.122
Facebook user type								
Active vs. passive^d^	-1.92	.65	[-3.18, -.65]	.003**	1.74	.38	[.99, 2.49]	< .001***
Instagram user type								
Active vs. passive^d^	-3.25	.81	[-4.83, -1.66]	< .001***	1.45	.49	[.49, 2.41]	.003**

^a^All models adjusted for participants’ gender, age, educational level, and employment status. ^b^Depressive symptomatology and self-esteem were treated as continuous numeric variables. ^c^*b*-coefficients and 95% CI of *b* retained from linear regression. ^d^Reference variable.

*p < .05. **p < .01. *** *p* < .001.

Daily Facebook use of two to five hours was associated with significantly higher self-esteem, after controlling for gender, age, educational level and employment status, *b* = 1.61, 95% CI [.78, 2.44], *p* < .001. The model explained 2% (adjusted 1%) of the variance in depressive symptoms (*R*^2^ = .02). Daily use of YouTube showed non-significant results regarding self-esteem.

Active Facebook use was significantly associated with lower BDI-II scores, after controlling for gender, age, educational level and employment status, *b* = -1.92, 95% CI [-3.18, -.65], *p* = .003. The model explained 2% (adjusted 1.5%) of the variance in depressive symptoms (*R*^2^ = .02). Likewise, self-reported active Instagram use was related to significantly reduced depressive symptoms, after controlling for gender, age, educational level and employment status, *b* = -3.25, 95% CI [-4.83, -1.66], *p* < .001). The model explained 4% (adjusted 3.4%) of the variance in BDI-II scores (*R*^2^ = .04). Self-reported type of YouTube use showed non-significant results concerning depressive symptoms.

Active Facebook use was associated with significantly increased levels of self-esteem, after controlling for gender, age, educational level and employment status, *b* = 1.74, 95% CI [.99, 2.49], *p* < .001. The model explained 4% (adjusted 3.4%) of the variance in depressive symptoms (*R*^2^ = .04). Similar results were also found concerning active use of Instagram, after controlling for gender, age, educational level and employment status, *b* = 1.45, 95% CI [.49, 2.41], *p* = .003). The model explained 3% (adjusted 2.5%) of the variance in depressive symptoms (*R*^2^ = .03). Self-reported type of YouTube use showed non-significant results regarding self-esteem.

### Interaction Effect Analyses

YouTube daily use of more than five hours showed a stronger association with depressive symptoms for males than for females (*p* for interaction = .026).

## Discussion

The present study investigated the association of social media use with self-esteem and depressive symptomatology in young adults. According to the results, increased daily time spent in YouTube (more than five hours) showed a significant association with higher depressive symptoms, while daily use of Facebook between two and five hours was related to significantly increased self-esteem, after adjusting for gender, age, educational level and employment status. YouTube daily use of more than five hours showed a stronger association with depressive symptoms for males than for females. Additionally, self-reported active use of Facebook and Instagram were associated with significantly lower depressive symptoms and higher self-esteem as compared to passive use.

In accordance with previous findings from Greece (Drosos et al., 2015), YouTube and Facebook displayed the highest percentages of everyday involvement with regard to our sample. Moreover, in line with our first hypothesis and previous studies (e.g. Lin et al., 2016; Pantic et al., 2012), daily YouTube use of more than five hours was associated with increased depressive symptomatology. Recent research has suggested that YouTube, unlike Facebook and Instagram, has been linked to increased perceived information overload for users due to the great amount of available video content. In addition, information overload in social media has been associated with higher depressive symptoms overtime (Matthes et al., 2020). On the other hand, depression is often associated with social withdrawal (Girard et al., 2014), hence it is possible that individuals with high depressive symptomatology tend to use YouTube more in comparison with other platforms, as it encourages less interactive involvement (Burgess & Green, 2009).

We also found that the association of increased daily time use of YouTube with depressive symptoms was more pronounced in males than in females. Conversely, Twenge and Martin (2020) have recently indicated that the relationship between increased time of social media use and low levels of psychological well-being is stronger in females. Women appear to use social media more in order to sustain their existing relationships compared to men (Muscanell & Guadagno, 2012), which has been associated with higher self-esteem (Wilcox & Stephen, 2013). Therefore, female users could exhibit lower depressive symptomatology compared to male, given that self-esteem is negatively related to depressive symptoms (Conti et al., 2014). Additionally, men with increased depressive symptoms have been found to be more susceptible to internet overuse compared to women (Liang et al., 2016), which could also apply to social media use.

In contrast with our first hypothesis and recent studies (Bergagna & Tartaglia, 2018; Woods & Scott, 2016), our results showed that increased daily use of Facebook is significantly related to higher self-esteem, although the effect size is small. According to Walther’s hyperpersonal model of computer-mediated communication (Walther, 2007) and previous research (Gonzales & Hancock, 2011), selective self-presentation on Facebook can lead to higher self-awareness and, therefore, an increase in users’ self-esteem. Moreover, individuals focusing on close friendly relationships on social networks have exhibited higher levels of self-esteem (e.g. Wilcox & Stephen, 2013). A possible mechanism explaining this relationship could be the positive feedback that users receive from their online friends, as it has been related to increased self-esteem levels (Valkenburg et al., 2017; Valkenburg et al., 2006).

This study also indicated that self-reported active use of Facebook and Instagram are linked with significantly lower depressive symptoms and higher self-esteem compared to passive use. These results correspond to recent findings (Escobar-Viera et al., 2018; Verduyn et al., 2015) and align with our second hypothesis. According to previous research, passive use of social networks has been related to feelings of envy and decreased life satisfaction (Krasnova et al., 2013), while envy on social media, such as Facebook, has significantly predicted depressive symptoms (Tandoc et al., 2015). On the other hand, higher self-esteem has been linked with increased life satisfaction (Moksnes & Espnes, 2013), and decreased feelings of envy (Vrabel et al., 2018). Thus, it appears that social comparison as a mechanism might provide explanation concerning our findings, since it could induce feelings of envy, while being related to passive activity in social media (Rozgonjuk et al., 2019). Furthermore, it is possible that high self-esteem encourages active behaviour in social media, as previous data has suggested a significant association between decreased feelings of self-worth and passive activities on platforms such as Facebook (Tazghini & Siedlecki, 2013).

### Strengths and Limitations

To our knowledge, the present study was one of the few focusing on the investigation of the association between social media use and mental health in a Greek sample of young adults. The large sample size along with the equal distribution of men and women provided adequate power to detect small effects. Additionally, self-esteem and depressive symptomatology were measured via standardised, valid and reliable psychometric tools displaying good psychometric properties with regard to our sample. Finally, the simultaneous assessment of different social networking platforms, instead of examining social media as a whole or focusing exclusively on a specific platform, was an additional strength of this study. Our fine-grained assessment of multiple platforms likely improved our measurement of overall frequency of social media use.

We acknowledge that there are also some limitations in our study. Given the cross-sectional design of the study, we are not able to establish the direction of the observed associations. Furthermore, even though both university students from various academic departments and non-university students from different regions in Greece were included in our study, generalizability in the Greek population may be limited. Moreover, due to the lack of a standardised Greek scale assessing social media use we used three items with specific artificial categories to measure daily time of social media involvement, which could be a noteworthy limitation as well. A number of methodological studies highlight a substantial loss of information as well as biased estimates when a continuous measure is broken up in artificial categories. It is also important to note that there are many different types of interactions that can be observed over social media, and our study assessed only overall time spent and type of use (active vs. passive) to social media sites. The type of social media use in terms of activity/passivity was examined only through self-reported questions, which could have relied our results and deductions exclusively on participants’ understanding of the terms “passive use” and “active use”. Additionally, the assessment of self-esteem and depressive symptomatology through self-reported scales instead of interviewing techniques, combined with our focus on non-clinical population, could restrict the possible clinical extensions of our findings. Furthermore, the small effect size reported was obtained in a sample of the general population which is expected to underestimate the effect size expected to occur in a clinical sample comprising persons displaying higher variability in self-reported symptom scales such as the BDI-II and RSES, and are in principle more vulnerable to social stressors. Additionally, the results from the multivariate linear regression analyses should be interpreted with caution given that the explained variance ranges between 1% and 4%; thus, if reported the other way around 96% to 99% of variance is not explained by the predictors in that model. Finally, although in our regression models we were able to adjust for a large number of confounding factors, because of the observational study design, residual confounding of other unmeasured confounders such as home environment or negative life events may still occur.

### Conclusion

The present study showed that there is a significant association between social media use and young adults’ mental health in terms of self-esteem and depressive symptomatology. Overall, our results add strength to previous research and could contribute to a deeper understanding of the association between social networks and human behaviour. However, a longitudinal investigation of this association is required to fully understand the temporal relationships aiding early identification of youth at risk and thus effective management of the social media use that lead to negative outcomes in mental health. In addition, future research could further explore gender differences concerning the relationship between social networking and young adults’ mental health. Moreover, upcoming studies could investigate the potential moderating or mediating effect of different patterns of use (e.g. passive and active involvement) on the relationship between time of social media use and mental health.
